# Intensity and duration of neutropenia relates to the development of oral mucositis but not odontogenic infection during chemotherapy for hematological malignancy

**DOI:** 10.1371/journal.pone.0182021

**Published:** 2017-07-27

**Authors:** Megumi Kishimoto, Masaya Akashi, Kazuyuki Tsuji, Junya Kusumoto, Shungo Furudoi, Yasuyuki Shibuya, Yumiko Inui, Kimikazu Yakushijin, Shinichiro Kawamoto, Atsuo Okamura, Hiroshi Matsuoka, Takahide Komori

**Affiliations:** 1 Department of Oral and Maxillofacial Surgery, Kobe University Graduate School of Medicine, Kobe, Japan; 2 Department of Oral and Maxillofacial Surgery, Nagoya City University Graduate School of Medical Sciences, Nagoya, Japan; 3 Department of Medical Oncology/Hematology, Department of Medicine, Kobe University Graduate School of Medicine, Kobe, Japan; 4 Division of Medical Oncology/Hematology, Kakogawa West City Hospital, Kakogawa, Japan; Texas Technical University Health Sciences Center, UNITED STATES

## Abstract

**Background:**

D-index which combines the intensity and duration of neutropenia is reported as a tool for evaluating the dynamics of neutropenia. This study aimed to analyze the relationship between D-index and oral complications (i.e., oral mucositis [OM] and odontogenic infection [OI]) during chemotherapies for hematological malignancies.

**Methods:**

A total of 421 chemotherapeutic courses in 104 patients were analyzed. Chemotherapeutic courses in patients who finished all of the prophylactic dental treatments were defined as “treatment Finish”. Chemotherapeutic courses in patients who did not finish prophylactic dental treatments were defined as “treatment not-Finish”. OM was evaluated according to the Common Terminology Criteria for Adverse Events, version 4.0. D-index was compared between chemotherapeutic courses with versus without oral complications.

**Results:**

D-index was significantly higher in chemotherapeutic courses with grade 1 or 2 OM (p < 0.001) than courses without OM. In contrast, higher D-index did not relate to the development of OI (p = 0.18). The occurrence of OI (p < 0.001) but not OM (p = 0.56) during chemotherapy was significantly higher in chemotherapeutic courses without the completion of dental intervention.

**Conclusions:**

Higher D-index relates to the development of OM. In contrast, OI occurs due to untreated odontogenic foci, and its occurrence does not relate to higher D-index.

## Introduction

Chemotherapy for hematological malignancies results in myelosuppression and increase the susceptibility of patients to severe infections. All odontogenic foci that are potential sources of systemic infection should be eliminated by prophylactic dental treatment before start of chemotherapy [1,2]. Therefore, dental oncologists have important roles in managing the oral health of these patients [3–5].

At first, we established a simplified grading of myelosuppressive intensity of various chemotherapy regimens that should facilitate the sharing of information between hematologists, dentists, and oral hygienists [6]. The necessity of effective communication between dental and medical providers was noted in the recent literature review [7]. And then, we formulated a novel protocol for dental intervention by modifying a previous protocol [8] for patients who underwent hematopoietic stem cell transplantation (HSCT), and reported the validity of our dental intervention protocol [9]. In our previous reports, we found that the period around first chemotherapy for de novo hematological malignancy patients was risky phase of the development of odontogenic infections due to instability in the immune system caused by the myelosuppressive chemotherapy and the untreated hematological tumor [6].

Neutropenia is one of major risk factors for infection in hematological malignancy patients undergoing chemotherapy. D-index, originally developed by Portugal et al. [10], uses data from white blood cell counts. This index may be helpful in defining different risks for invasive mold infections in neutropenic patients [10]. Calculation of the D-index was based on plotting the absolute neutrophil counts over the course of the episode of neutropenia in a graph. The area under the curve (AUC) of the neutrophil count can combine the duration and intensity of neutropenia. However, the AUC is not adequate for clinical use because patients who have a long duration of severe neutropenia have similar AUCs as patients with short, but not severe, neutropenia. In contrast, the D-index geometrically represents the area over the neutrophil curve and expresses the neutrophil deficit. The calculated value of D-index means only the intensity and duration of neutropenia obtained at the end of the episode of neutropenia, therefore, does not serve as a predictor of infectious complications [10]. A cumulative D-index (c-D-index), defined as the cumulative index from the start of neutropenia until the date of the first clinical manifestation of infection, could be used for the real-time assessment of the risk for infection [11]. Previous studies show that the c-D-index has a high negative predictive value for mold infection in leukemia patients who underwent chemotherapy and for pulmonary infection in recipients of HSCT [11].

This study aimed to analyze the relationship between the D-index and oral complications (i.e., oral mucositis [OM] and odontogenic infection [OI]) during myelosuppressive chemotherapy.

## Materials and methods

### Patients

A total of 104 consecutive adult patients who underwent chemotherapy, including HSCT, at Kobe University Hospital between October 2012 and March 2014 were included in this prospective study. A subclass analysis focusing on the D-index was performed in patients who were prospectively enrolled in our recent study [9] and newly added. The ethics committee of Kobe University approved this study (No. 1325, 2012). Written informed consent was obtained from all patients.

### Grouping of the patients

In each case, the hematologist informed the dentist of the patient’s diagnosis, the day on which chemotherapy was begun, and the myelosuppression grade of chemotherapy. The details of our myelosuppression grading system has been previously described [6,9]. In brief, the myelosuppressive intensity of grade A chemotherapies, including some oral agents and infusions mostly performed for outpatients (e.g., Rituximab alone for malignant lymphoma; BD and MP for multiple myeloma), was mild. The myelosuppressive intensity of grade B chemotherapies, including many different regimens (e.g., consolidation regimens for leukemia; ABVD, CHOP, and ESHAP for malignant lymphoma; azacytidine for myelodysplastic syndrome), was moderate. The myelosuppressive intensity of grade C chemotherapies, which included remission induction therapy for acute leukemia, high-dose chemotherapy with peripheral blood stem cell harvest, and salvage chemotherapy for NK/T cell lymphoma was severe. Conditioning regimens for HSCT, which cause the most severe myelosuppression and persistent immunodeficiency (known as myeloablative conditioning regimens) were classified as grade D. In this study, reduced-intensity conditioning [12] was also included in grade D chemotherapy.

The attending dentists (M.K. or K.T.) evaluated oral condition before the initiation of chemotherapy for hematological malignancy by medical inquiry, intraoral examination (inspection, palpation, and periodontal examination), and panoramic x-ray images in all patients.

The observation was performed from the initiation day of chemotherapy until the day before starting the next round of chemotherapy. Patients in whom oral complications occurred were recorded. When same patients received chemotherapy with the same regimen repeatedly, each course was evaluated as a single observation period. Each observation period was classified into two groups. The “treatment Finish” group included patients who finished all of the dental treatments according to our dental intervention protocol before the initiation of chemotherapy. The “treatment not-Finish” group included patients who did not finish all dental treatment according to our dental protocol before chemotherapy, because the chemotherapy should be immediately initiated owing to the state of tumor. After completing dental treatment following the end of chemotherapy cycle, “treatment not-Finish” patients were transferred to the “treatment Finish” group. Our protocol for dental interventions for patients with hematological malignancies has previously been described in detail [9], and is shown in S1 Fig. In temporary dental filling was undergone for enamel or dentine caries. Pulpitis with acute symptoms such as spontaneous pain was treated with pulpectomy, canal filling with calcium hydroxide paste containing iodoform, and temporary sealing. Teeth with apical periodontitis (periapical radiolucency larger than 5 mm) were extracted. Teeth with apical periodontitis (periapical radiolucency of smaller than 5 mm) and acute symptoms such as spontaneous pain, obvious percussion pain or redness, swelling, tenderness, or pus drainage of the surrounding gingiva were also extracted. Teeth with marginal periodontitis with (1) probing depth deeper than 8 mm, (2) sever mobility (i.e., ability to depress the tooth in a vertical direction), (3) acute symptoms as mentioned above or past history of repeated acute inflammation were extracted. In cases of pericoronitis, totally or partially impacted teeth with acute symptoms as mentioned above or past history of repeated acute inflammation were extracted. All patients received routine oral management by their dentists or dental hygienists with reference to maintaining a plaque score less than 20% [13].

Each observation period was also divided into two groups: “First Chemo” and “not-First Chemo” group. “First Chemo” group included de novo patients who received chemotherapy for the first time, and “not-First Chemo” group consisted of patients who had previously received chemotherapy. The occurrence of oral complications was compared between each group.

### Clinical findings

Oral complications during chemotherapy included OM and OI. OM was evaluated according to the Common Terminology Criteria for Adverse Events, version 4.0. OI was defined the marginal periodontitis, apical periodontitis, and pericoronitis with acute symptoms as mentioned above. OI also included redness, warmth, swelling, pus discharge, pain in or around a tooth or periapical/periodontal tissues, and trismus (< 20 mm) caused by dental disease.

**Fig 1:**
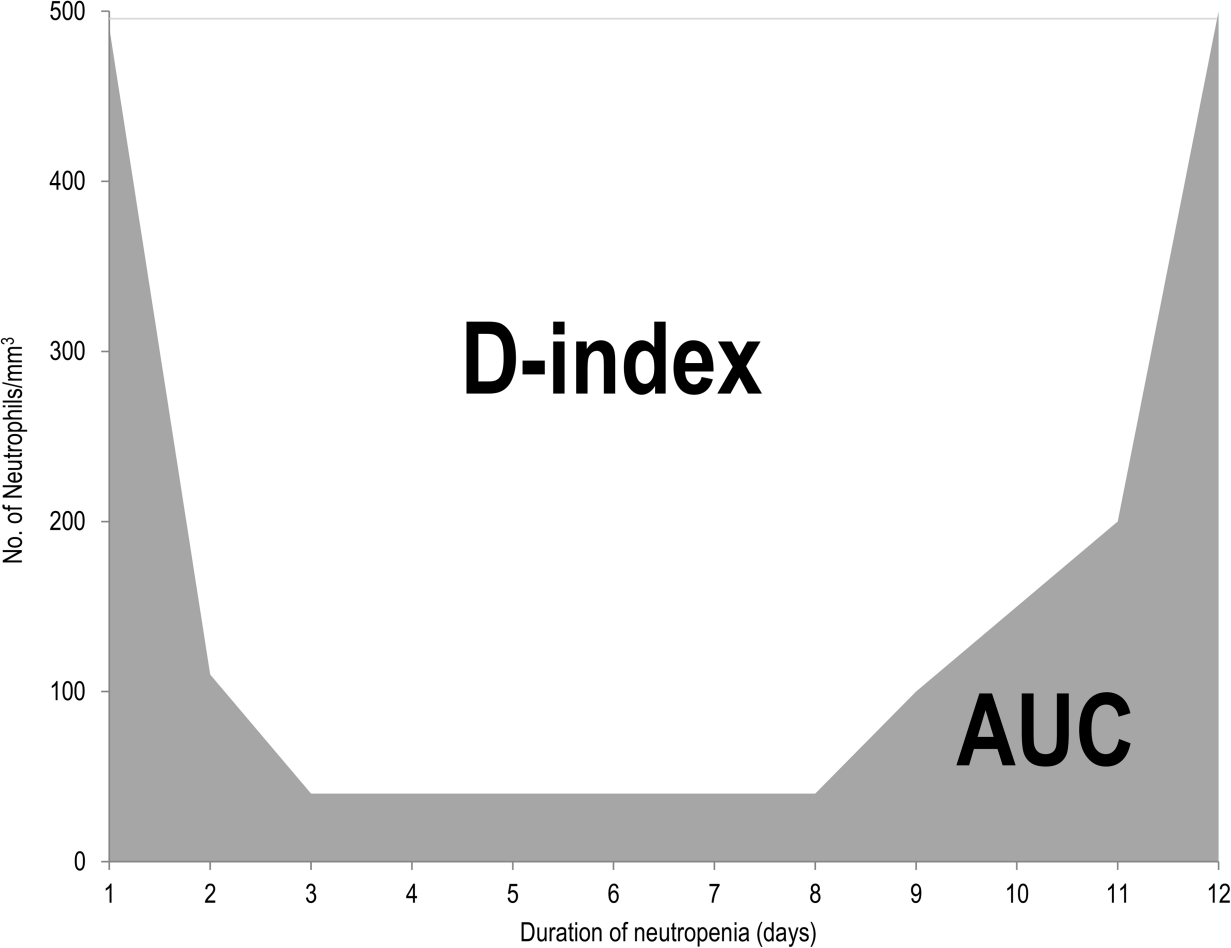
Area over the neutrophil curve (D-index). White area indicates D-index. AUC, area under the curve. D-index was originally developed by Portugal et al [10].

To calculate the D-index, the number of white blood cell during the whole duration of neutropenia was counted. The D-index was calculated as the difference between AUC and expected neutrophil area (i.e., 500/μL x days with neutropenia) if patients did not develop neutropenia [11] (Fig 1). The date of the first clinical manifestation of oral complications was recorded in medical charts, and the c-D-index was also calculated.

### Statistical analysis

Statistical analyses (Fisher’s exact test, χ^2^ test, Mann–Whitney U test, and Kruskal-Wallis test with Bonferroni correction) were performed using R software (R Development Core Team, 2011). A value of p < 0.05 was regarded as indicating statistical significance.

## Results

There was a total of 421 observation periods for the 104 patients (63 men, 41 women). The median age of the patients was 61.0 years (range, 20–85 years). The diagnosis of hematological malignancies is shown in Table 1. The myelosuppression grades of chemotherapy regimens encountered in this study were A in 32 courses (8%), B 313 (74%), C 46 (11%), and D 30 (7%).

**Table 1:** Relationship between type of hematological malignancy and oral complications.

Diagnosis	Number of patients n = 104: male/female	Number of grade B–D chemotherapy courses n = 389	Oral mucositis n = 67	Odontogenic infection n = 34
Acute myeloid leukemia	18:13/5	56	17	12
Acute lymphoblastic leukemia	11:7/4	54	17	3
Myelodysplastic syndrome	7:5/2	16	4	1
Chronic myeloid leukemia	1:1/0	4	0	0
Diffuse large B-cell lymphoma	34:18/16	169	12	16
Follicular lymphoma	6:2/4	25	2	0
Peripheral T-cell lymphoma	5:4/1	15	2	2
Hodgkin’s lymphoma	3:2/1	15	1	0
Adult T-cell leukemia/lymphoma	3:3/0	9	4	0
Multiple myeloma	7:1/6	0	0	0
Others	9:6/3	26	8	0

**Fig 2:**
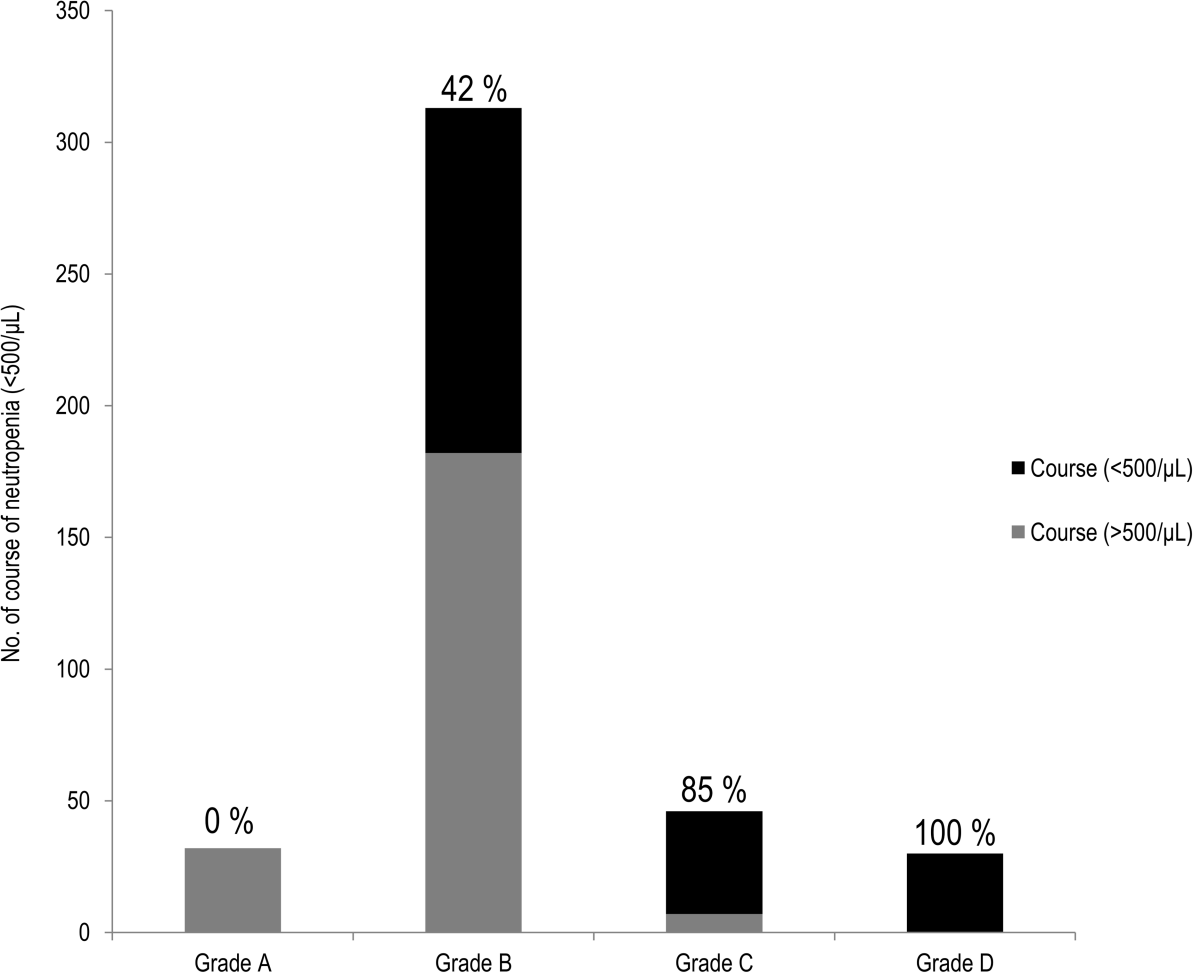
The number of courses with neutropenia in each grade of chemotherapy.

**Fig 3:**
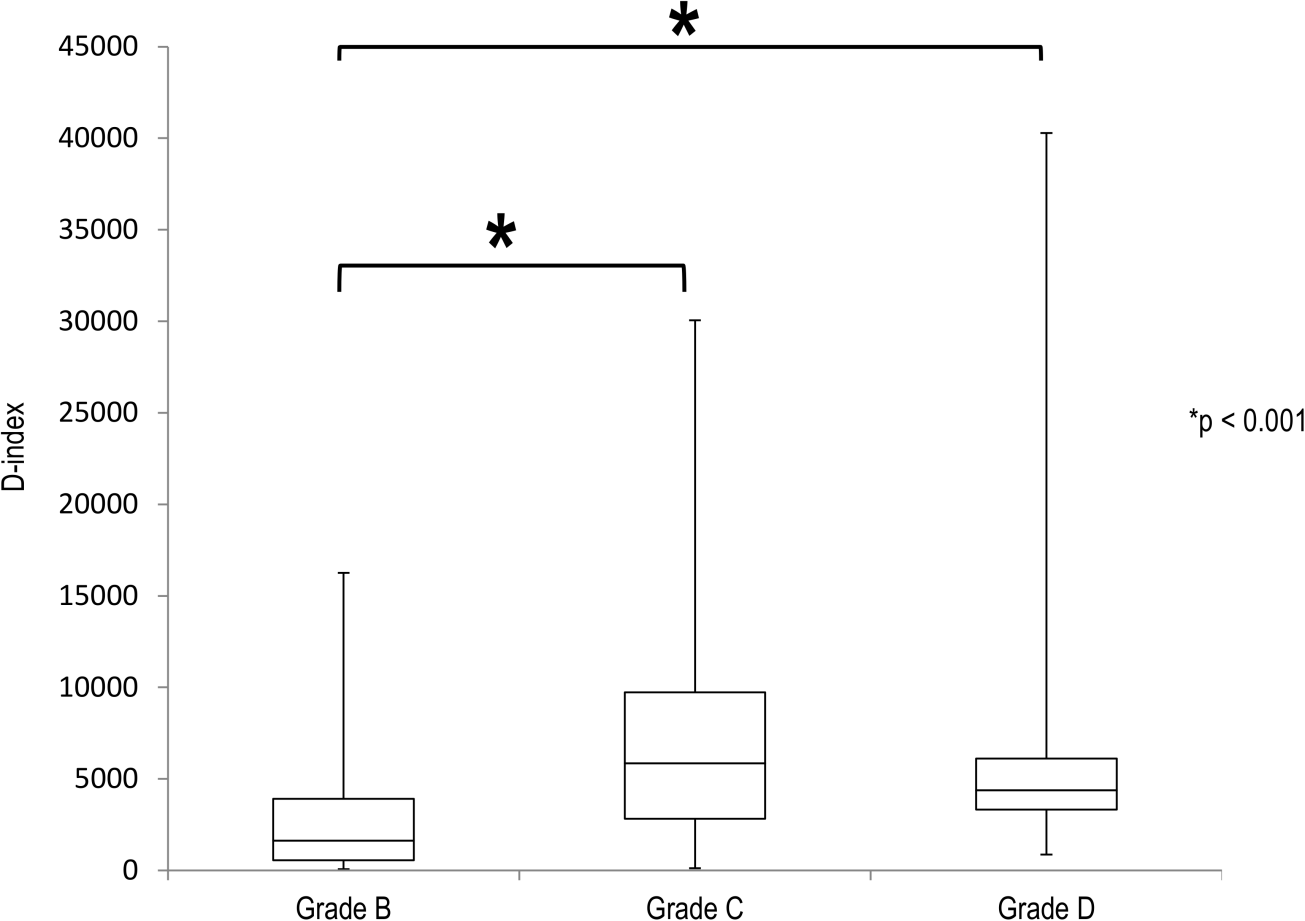
Box plot of the D-index in each grade of chemotherapy.

The validity of our myelosuppression grading system was assessed. None of patients who received grade A chemotherapy had neutropenia (< 500/μL), and the numbers of courses with neutropenia increased in association with the severity of myelosuppression (Fig 2). There were no OM and OI during grade A chemotherapy. The values of the D-index of grade C (p < 0.001) and D (p < 0.001) were significantly higher than grade B chemotherapy (Fig 3).

The occurrence of oral complications during chemotherapy except for grade A (i.e., the number of observation periods were 389) was assessed. As shown in Table 1, all patients with multiple myeloma underwent grade A chemotherapy. No significant relationship was found between the type of hematological malignancy and the occurrence of oral complications (Table 1). The oral complications were observed in 93/389 chemotherapeutic courses (23.9%). OM was observed in 67/389 courses (17.2%), and OI 34/389 courses (8.7%). In 8/389 courses (2.1%), both OM and OI were observed. Grade 1 OM was observed in 44 courses (65.7%) and grade 2 in 23 (34.3%). Grade 3, 4, and 5 OM were not found in this study. The site of OI was maxilla in 19 courses (55.9%), mandible 12 (35.3%), and both 3 (8.8%). OI frequently occurred in molar region as follows: 17 (50%) in molar, 9 (26.5%) in wisdom tooth, 6 (17.6%) in anterior region, and 2 (5.9%) in both anterior and posterior regions. The etiology of OI was marginal periodontitis in 15 (44%), apical periodontitis in 11 (32%), and pericoronitis in 8 (24%), respectively.

The value of the D-index was significantly higher in courses with OM (p < 0.001). In contrast, there was no significant difference between courses with versus without OI (p = 0.18) (Table 2).

**Table 2:** Relationship between D-index and oral complications.

	n = 389 (%)	D-index, median (range)	p value
Oral mucositis			
Yes	67 (17.2)	3105 (0–40275)	<0.001
No	322 (82.8)	0 (0–30059)	
Odontogenic infection			
Yes	34 (8.7)	518 (0–16250)	0.18
No	355 (91.3)	117 (0–40275)	

The occurrence of OM (p < 0.001) but not OI (p = 0.11) significantly increased according to the severity of myelosuppression (Table 3). The occurrence of OI (p < 0.001) but not OM (p = 0.56) was significantly higher in “treatment not-Finish” group than “treatment Finish” group (Table 3). The occurrence of OM (p < 0.001) was significantly higher during “First” than “not-First” chemotherapy (Table 3). There were no significant differences of the value of D-index during courses with oral complications between “treatment Finish” and “treatment not-Finish” groups (OM, p = 0.67; OI, p = 0.15) (Table 4). In contrast, the value of c-D-index was significantly higher in “treatment Finish” group than “treatment not-Finish” group only in courses with OI (p = 0.037) (Table 4).

**Table 3:** Relationship between parameters and oral complications.

Parameters	n = 389			
	Oral mucositis n = 67 (%)	p value	Odontogenic infection n = 34 (%)	p value
Myelosuppression grade		<0.001		0.11
B	39/313 (12.5)		24/313 (7.7)	
C	13/46 (28.2)		8/46 (17.4)	
D	15/30 (50)		2/30 (6.7)	
Dental intervention		0.56		<0.001
Finish	45/273 (16.5)		4/273 (1.5)	
not-Finish	22/116 (19.0)		30/116 (25.9)	
Chemotherapy		<0.001		0.06
First	40/134 (30.0)		17/134 (12.7)	
not-First	27/255 (10.6)		17/255 (6.7)	

**Table 4:** Relationship between c-D-index and oral complications.

	Oral mucositis n = 67		Odontogenic infection n = 34	
	D-index, median (range)			
Dental intervention		p value		p value
Finish	2835 (0–40275) (n = 46)	0.67	4736 (329–7101) (n = 4)	0.15
not-Finish	3372 (0–16250) (n = 21)		344 (0–16250) (n = 30)	
	c-D-index, median (range)			
Dental intervention		p value		p value
Finish	894 (0–39759) (n = 46)	0.41	3321 (329–5579) (n = 4)	0.037
not-Finish	1487 (0–12952) (n = 21)		0 (0–12810) (n = 30)	

## Discussion

This is a novel study aiming to analyze the relationship between the D-index and oral complications during myelosuppressive chemotherapies. We found that higher values of the D-index during chemotherapy causing severe myelosuppression were related to the higher occurrence of OM but not OI.

The D-indexproposed is associated with invasive mold infections [10]. However, they noted some limitations in their study, one of which was that their data were applicable only to patients with acute myeloid leukemia in induction remission [10]. They mentioned that assessing the typical D-index of different chemotherapy regimens may help to define strategies for monitoring and using antifungal agents [10].

In the current study, no patients who received grade A chemotherapy had neutropenia, and a significant difference in the D-index was found among myelosuppressive grades (between grades B and C, and B and D). These results reflected the validity of our myelosuppression grading of chemotherapy. We also found that the incidence of OI was significantly higher in the “treatment not-Finish” than “treatment Finish” groups during myelosuppressive chemotherapy. This result indicated the validity of our dental intervention protocol. Importantly, the occurrence of OI did not relate to the myelosuppression grade (Table 3), indicating that OI can occur mostly due to untreated odontogenic foci during any myelosuppressive chemotherapy.

This study showed that the higher D-index relates to the occurrence of OM. However, the severity of OM observed in this study was only mild to moderate. We have considered that the period around the first chemotherapy for de novo hematological malignancy patient is the risky phase of OI. Notably, this study indicated the possibility that the period around the first chemotherapy was also the risky phase of developing OM (Table 3). Although no significant relationship between the type of hematological malignancy and the occurrence of oral complications was found in this study, the diverse biological behaviors of each hematological malignancy (e.g., the suppression of B cell function in multiple myeloma) should be noted.

The occurrence of OM did not relate to the completion of dental intervention in this study (Table 3). In the recent study about patients who underwent superselective intra-arterial chemotherapy concurrent with radiotherapy for oral cancer, the professional oral health care reduced the total dose of morphine for the pain control and shortened the hospital days, indicating the utility of professional oral care [14]. In the recent randomized controlled pilot study in hospitalized patients with acute myeloid leukemia awaiting HSCT, the incidence of OM was 100%, and the highest OM grade was lower in strict oral care self-management support protocol group (OM grade 2) than in usual care group (OM grade 3) [15]. OM durations were shorter in oral care support protocol group than in usual care group, and OM onset was also later in oral care protocol group than usual care group [15]. To reduce oral complications, not only dental intervention by dental oncologists but also strict self-management may be necessary.

We also calculated c-D-index, defined as the cumulative D-index from the start of neutropenia until the development of infection [10]. A c-D-index could be used for the real-time assessment of the risk of infection [11]. A previous study reported that the c-D-index might determine the risk of pulmonary infection in consolidation chemotherapy [11]. Kimura et al [11] concluded the necessity of a clinical trial of c-D-index-guided preemptive antifungal therapy. Recently, the same authors showed that c-D-index with a cut-off value had predictive value for the development of pulmonary infection [16]. The current study shows that OM develops depending on the intensity of duration of neutropenia, irrespective of the completion of dental intervention. In contrast, the development of OI depends on the incompletion of dental intervention. Interestingly, we found that the value of c-D-index in the group developing OI despite the completion of dental intervention was significantly higher than in the group developing OI in which dental intervention was not completed. This result seems to indicate the presence of OI caused by the neutropenia despite the completion of dental intervention. Prevention of such OI is our future task.

There are some limitations of this study. First, this study included only a small sample size. Second, we calculated c-D-index, but could not identify the cut-off value of c-D-index. Third, in our previous study, systemic findings including body temperature, respiratory symptoms, C-reactive protein level, and blood culture results were evaluated [9]. The current study did not evaluate systemic findings. Therefore, we are planning a prospective study with a larger number of subjects to overcome these limitations.

## Conclusion

The higher D-index related to the occurrence of OM but not OI. The occurrence of OI related to the incompletion of dental intervention, rather than the severity of myelosuppression caused by chemotherapy.

## Supporting information

S1 Fig Dental protocol for hematological malignancy patients, originally described by Tsuji et al [9]. (TIF)
