# Supplementary figures and images for: Intensity and duration of neutropenia relates to the development of oral mucositis but not odontogenic infection during chemotherapy for hematological malignancy
Source: PLoS One. 2017 Jul 27;12(7):e0182021. doi: 10.1371/journal.pone.0182021 (PMC5531589; doi:10.1371/journal.pone.0182021)

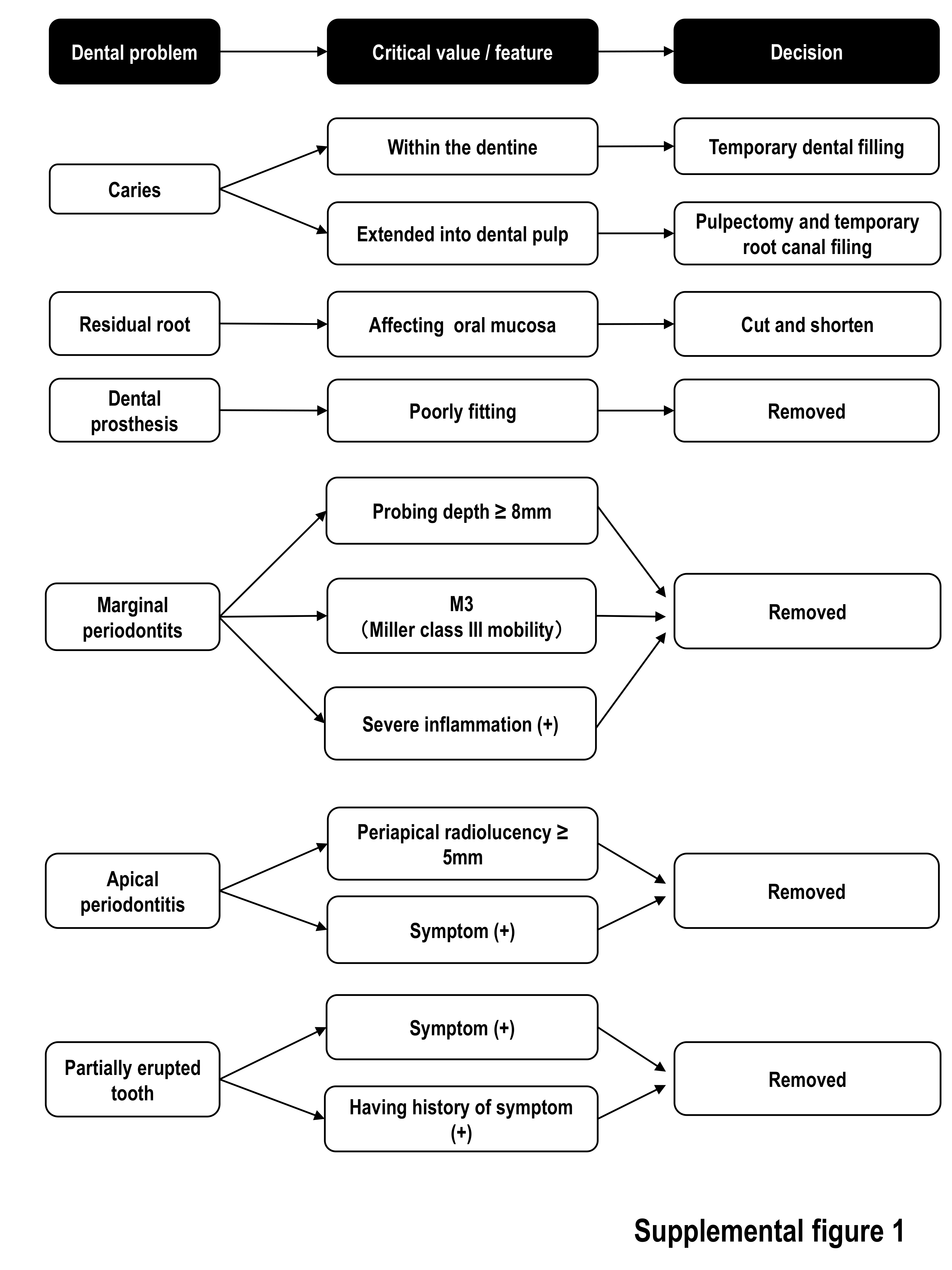

Supplement: S1 Fig — (TIF) [file pone.0182021.s001.tif]
